# Supplementary material for: From prototype to outbreak: conserved pathogenesis of Oropouche virus in a novel murine pregnancy model highlights its public health implications
Source: bioRxiv. 2025 Aug 2:2025.08.02.668287. Preprint. [Version 1] doi: 10.1101/2025.08.02.668287 (PMC12324540; doi:10.1101/2025.08.02.668287)
Supplement: 1 — Supplemental Figure 1. rOROVMZsG in immunocompetent C57BL/6J mice. (a) Schematic of experimental design. WT C57BL/6J mice were infected SC with either rOROVMZsG (3 groups, each n=3) or UV-inactivated rOROVMZsG (n=3), and euthanized at 5, 7, or 14 dpi. (b) Percent weight change of mice from baseline compared to UV-inactivated virus controls. (c) vRNA loads per gram of tissue in the liver, spleen, heart, lung, and brain of rOROVMZsG or UV-inactivated infected mice as measured by RT-qPCR. The dashed line represents the limit of detection, ND = not detected. (d) Representative fluorescence images from virus isolations in Vero E6 cells showing infectious rOROVMZsG in homogenized liver and spleen samples from infected mice (EVOS M5000 imaging system, ThermoFisher). Supplemental Figure 2. Infection with rOROV BeAn19991 causes liver pathology and placental infection. (a) Representative images of fetuses harvested from early or mid-gestation infected dams, showing normal gross morphology. (b) Detection of replication-competent virus in Vero E6 cells infected with placental homogenates. Immunofluorescence staining shows rOROV (red) at 24 hpi. Nuclei stained with DAPI (blue). Four representative placental isolates (#P1–P4) are shown. (c) Representative H&E-stained liver sections from mock- and rOROV-infected pregnant C57BL/6J dams. (d) vRNA levels in the liver and spleen of pregnant mice infected at mid-gestation with OROV 240023 (e) vRNA levels in matched placentas and fetuses following mid-gestation infection. The dashed line represents the limit of detection. ND = not detected. Supplemental Figure 3. Optimization of AM0059/88 M segment minigenome assay. BSRT7/5 cells were co-transfected with either increasing amounts of pTM1-N and constant amounts of pTM1-RdRp (250 ng) or increasing amounts of pTM1-RdRp and constant amounts of pTM1-N (250 ng), along with an AM0059/88 M-segment minigenome encoding humanized Renilla luciferase (hRenilla). A firefly luciferase-expressing plasm [file NIHPP2025.08.02.668287V1-supplement-1.pdf]

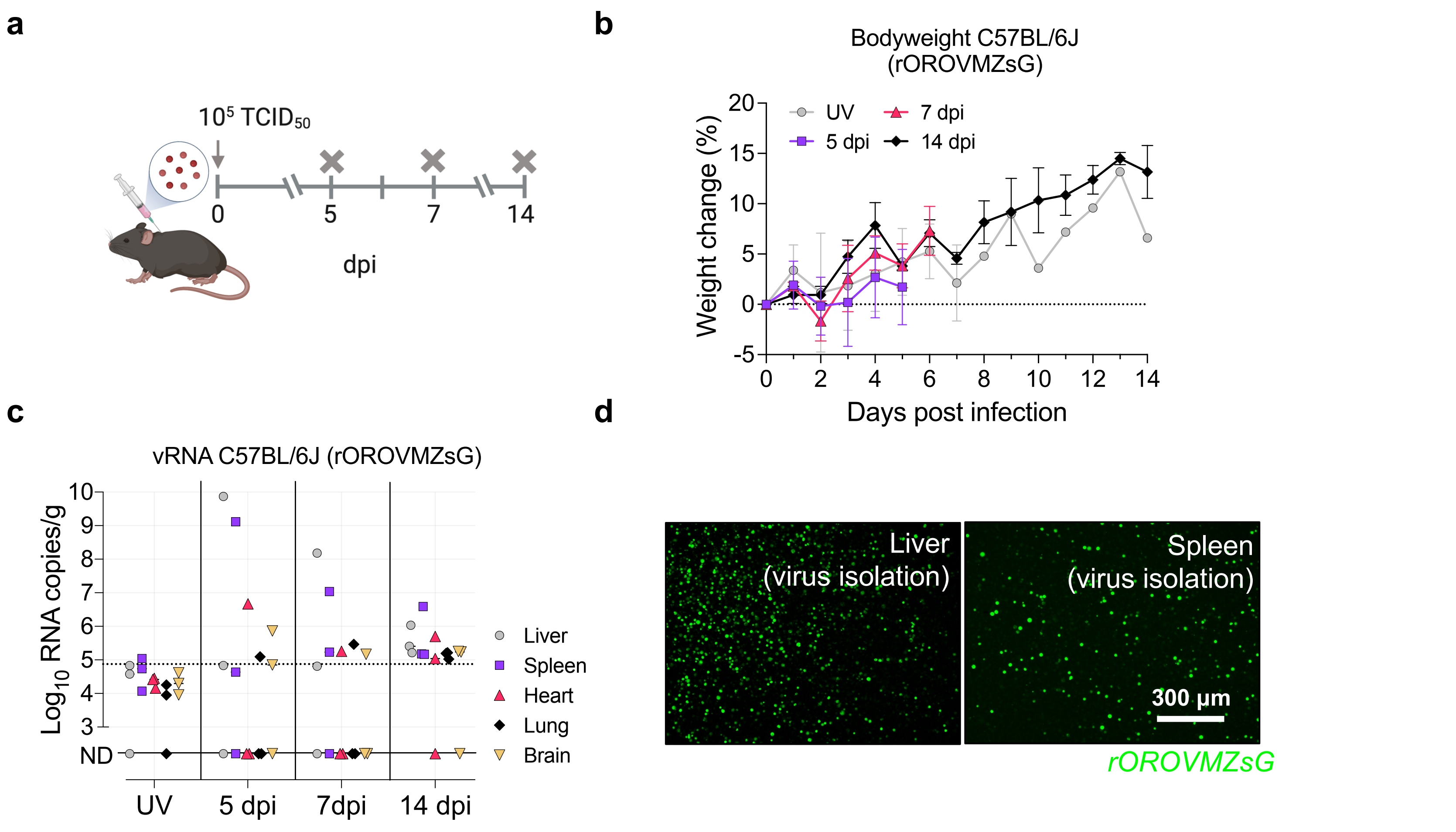

Supplemental Figure 1

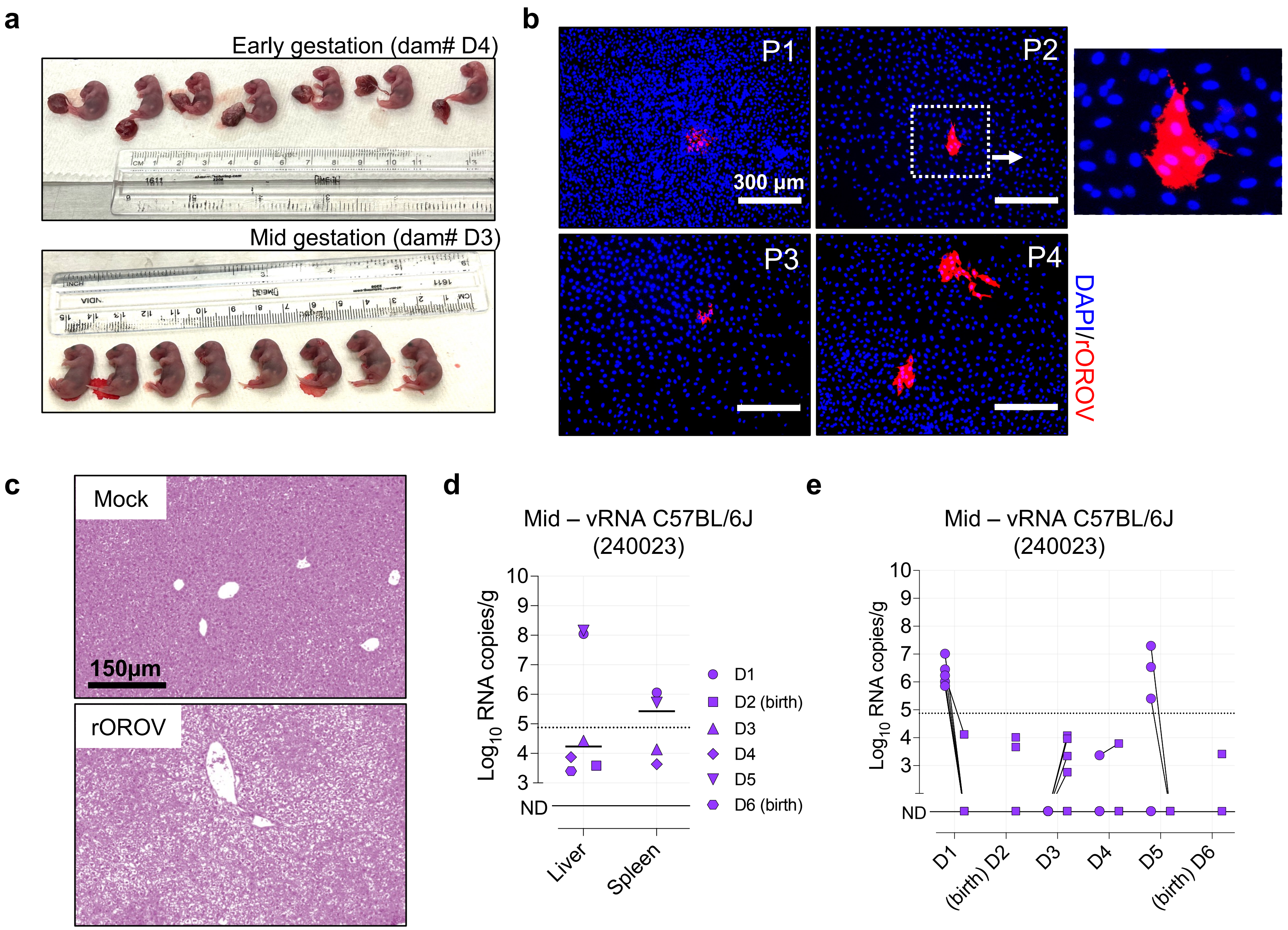

Supplemental Figure 2

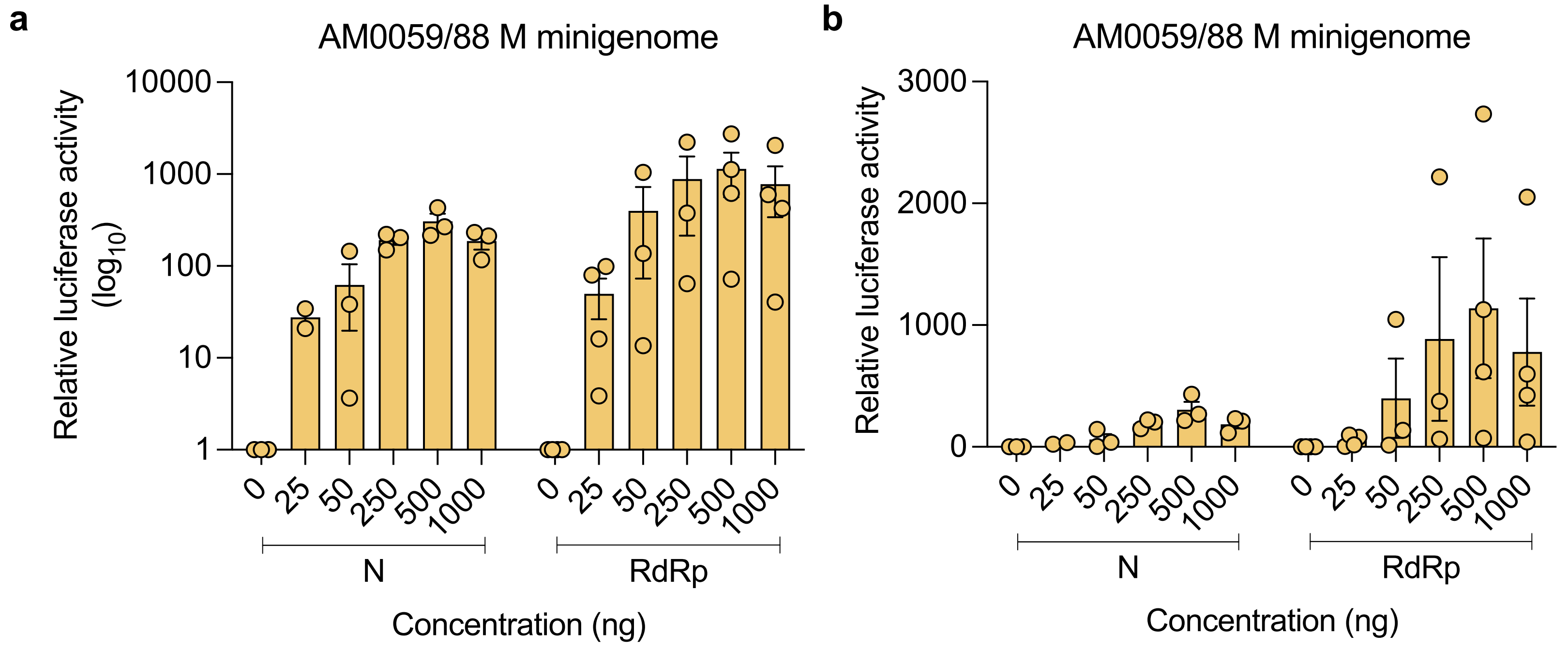

Supplemental Figure 3

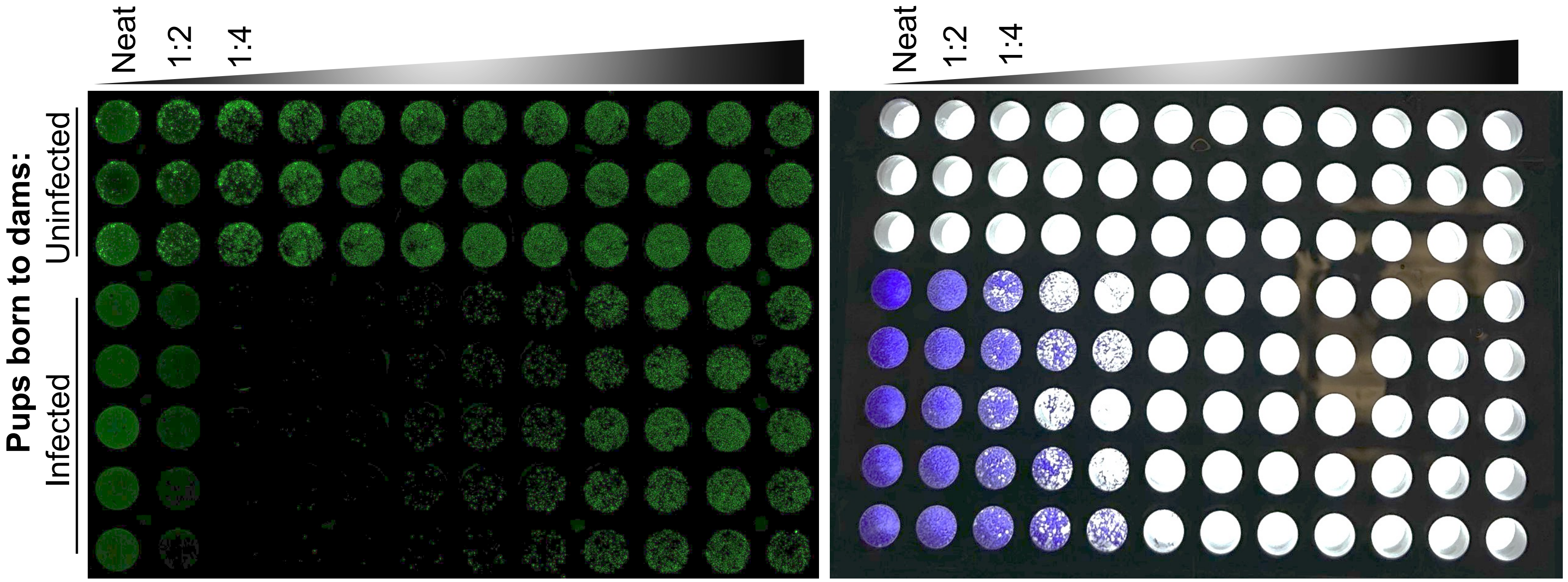

Supplemental Figure 4

Supplemental Table: HCR probes used in this study.

Pair #	Initiator	Spacer	Probe	Probe	Spacer	Initiator
1	GTCCCTGCCTCTAT ATCT	tt	TACATCTACATTTGATC CGGAGGCA	CATTTTCAACGATGTACCA CAACGG	tt	CCACTCAACTTTAACC CG
2	GTCCCTGCCTCTAT ATCT	tt	CAAGTGCTCAATGCTG GTGTTGTTA	TATGTGGCATTTGAAGCTA GATACG	tt	CCACTCAACTTTAACC CG
3	GTCCCTGCCTCTAT ATCT	tt	AAGATGTCTTACGTAAG ACATCGAG	TCTTCTTCCTCAACCAAAA GAAGGC	tt	CCACTCAACTTTAACC CG
4	GTCCCTGCCTCTAT ATCT	tt	CCAATTTGCAATGGTTA ATAACCAT	CATGGTTGACCTTACTTTT GGTGGG	tt	CCACTCAACTTTAACC CG
5	GTCCCTGCCTCTAT ATCT	tt	GACAACGGTCTTACCCT GCACCGTC	CCACAGTTCCAGTCGAAT CCAGTGC	tt	CCACTCAACTTTAACC CG
6	GTCCCTGCCTCTAT ATCT	tt	CCCAGATGCGATCACC AATTAAGCA	CAGGATACCTAGCGCGCT GGGCCTT	tt	CCACTCAACTTTAACC CG
7	GTCCCTGCCTCTAT ATCT	tt	TTTGGCTGAGGTAAAG GGCTGTACT	TGAGTTCAGAGCCACTGT AGTAGTG	tt	CCACTCAACTTTAACC CG
8	GTCCCTGCCTCTAT ATCT	tt	TTTGCTGCTGGTGCTGA GATGTTCC	AATGATGGTGACGCAATG TACCTGG	tt	CCACTCAACTTTAACC CG
9	GTCCCTGCCTCTAT ATCT	tt	TTGAGATGCATAGGGTT CTCAAGGA	AAACATTCACTTTCTTCCC TTTGGT	tt	CCACTCAACTTTAACC CG
10	GTCCCTGCCTCTAT ATCT	tt	CCTCCGGCAACGGTAT GGCCAAAAG	CATGGATGTCAACTTTATG AAGAAA	tt	CCACTCAACTTTAACC CG
11	GTCCCTGCCTCTAT ATCT	tt	GTTGCTGTAAGAGCAG CTTTTGAAG	GCCGAGCAATGGATGCGT GAAGAAA	tt	CCACTCAACTTTAACC CG
12	GTCCCTGCCTCTAT ATCT	tt	GATTCTCCCCAGCAGC AAGAGACTT	TAGGCACTCTGGCCTGGG CCAGAAC	tt	CCACTCAACTTTAACC CG
